# A Nomogram to Better Predict the In-Hospital Mortality of Trauma Patients with Sepsis in the Intensive Care Unit

**DOI:** 10.1155/2022/4134138

**Published:** 2022-09-09

**Authors:** Jing Qi, Qin Xie, Zhenzhou Li, Chuanzheng Sun

**Affiliations:** Department of Emergency, The Third Xiangya Hospital of Central South University, Changsha, Hunan, China

## Abstract

**Background:**

Trauma has a high incidence and mortality worldwide, and sepsis is one of the main causes of mortality in trauma patients. Therefore, it is essential to identify the risk factors of in-hospital mortality for trauma patients with sepsis.

**Methods:**

Data were extracted from the Medical Information Mart for Intensive Care III database and divided into a training set and internal validation set, and another Chinese dataset was used as external validation set. Then, risk factors were estimated using univariate and multivariate logistic regression analyses in the training set. Finally, a nomogram was created to predict the probability of in-hospital mortality for trauma patients with sepsis.

**Results:**

A total of 503 patients were enrolled in our study (335 in the training set and 168 in the validation set). Multivariate logistic regression analysis revealed that age (1.047 [1.025–1.071]), respiratory rate (1.258 [1.135–1.394]), PTT (1.026 [1.008–1.044]), ventilation (6.703 [1.528–29.408]), and vasopressor use (3.682 [1.502–9.025]) were independent factors associated with in-hospital mortality. The nomogram for trauma-related sepsis predicted in-hospital mortality with AUC values of 0.8939 in the training set, 0.8200 in the internal validation set, and 0.7779 in the external validation set.

**Conclusions:**

The new nomogram has a well predicted value for in-hospital mortality for patients with trauma and sepsis in intensive care units.

## 1. Introduction

To date, trauma still exerts a major burden on health-care resources worldwide. Trauma remains the leading cause of death among individuals younger than 45 years in the United States [1]. Trauma patients are especially prone to developing sepsis, which is associated with increased hospital length of stay and mortality [2]. Sepsis is defined as a life-threatening organ dysfunction caused by a dysregulated host response to infection [3]. Dysregulation of the host inflammatory response contributes to the mortality of patients with sepsis after trauma [4–6]. Complications after trauma, including sepsis, increase the probability of a poor prognosis and mortality in the intensive care unit [7].

Some studies have been conducted to explore the risk factors for post-traumatic sepsis, such as age, glycosylated hemoglobin, Injury Severity Score, number of injuries, number of red blood cell units transfused, and emergency surgery, which are characterized by high mortality [2, 8, 9]. A predictive score has been developed to predict sepsis risk among trauma patients [10]. A nationwide cohort study reported a relationship between sex differences and mortality in patients with sepsis after trauma [11]. In summary, recent research has predominantly focused on the risk factors for post-traumatic sepsis, and few studies have examined risk factors associated with the in-hospital mortality of trauma patients with sepsis. Sepsis is one of the leading causes of death in the later stage of trauma in intensive care units worldwide, and the latest data on the prognosis of trauma patients with sepsis are still insufficient. Therefore, it is necessary to build a reliable prediction model of the in-hospital mortality of trauma patients with sepsis, which might help us improve our understanding of the in-hospital mortality of trauma patients with sepsis and might provide an opportunity to decrease mortality.

Nomograms have been widely used to integrate multiple independent risk factors, quantify the impact of different factors and visualize results to predict disease prognosis [12, 13]. In our study, we aimed to identify risk factors based on routine variables that can be measured easily during clinical assessments within a few hours of admission and construct a nomogram associated with in-hospital mortality for trauma patients with sepsis, which might help improve outcomes with timely implementation of targeted interventions.

## 2. Materials and Methods

### 2.1. Data Collection

All the data in the current study were extracted from an online international database, Medical Information Mart for Intensive Care III (MIMIC III), which encompasses 53,243 different hospital admissions for adult patients in intensive care units between 2001 and 2012 [14]., and a dataset from a Chinese hospital which was conducted as external validation set [15]. All the patients in the database were deidentified for privacy protection purposes, and the need for informed consent was waived. Use of both databases was approved by the Institutional Review Boards of BIDMC and the Massachusetts Institute of Technology.

The inclusion criteria in the study included the following: (1) admission to the ICU for trauma, (2) age ≥18 years old, and (3) sepsis occurring after trauma. Patients with ICU lengths of stay shorter than 24 hours were excluded. For patients who were admitted to the ICU more than once, only the first ICU stay was considered. The definition of sepsis was provided by the Third International Consensus definitions for sepsis and septic shock (sepsis-3) [3]. The data were then randomly divided into a training set (2/3 of the data) and a validation set (remaining 1/3 of the data), and finally a Chinese dataset was conducted as an external validation set.

### 2.2. Demographic and Clinical Data

Demographic data were collected using structured query language (SQL) and included age, sex, weight (first day in ICU), and severity at admission as measured by the Glasgow Coma Scale (GCS), the Sequential Organ Failure Assessment (SOFA) score [16], the simplified acute physiology score II (SAPS-II) score [17] and the acute physiology and chronic health evaluation (APACHE-III) score [18]. The comorbidities of the included patients were collected and included coronary heart disease (CHD), hypertension, chronic obstructive pulmonary disease (COPD), and diabetes. All comorbidities were diagnosed based on the relevant ICD-9 codes in the database. Vital signs, including temperature, heart rate, respiratory rate, and mean arterial pressure (MAP), were also recorded at 24 h after ICU admission. An average was used when more than one record was available.

### 2.3. Laboratory Data and Interventions

All variables were recorded within 24 h after the patient was admitted to the ICU. Laboratory data included white blood cell (WBC) count, red blood cell count, hemoglobin count, hematocrit, platelet count, arterial blood gas results (including lactate and glucose), blood urea nitrogen (BUN), blood creatinine, sodium, potassium, calcium, magnesium, chloride, phosphorous, partial thromboplastin time (PTT), prothrombin time (PT), and the international normalized ratio (INR). The interventions included continuous renal replacement therapy (CRRT), ventilation and use of vasopressors (including dobutamine, dopamine, epinephrine, isoproterenol, norepinephrine, phenylephrine and other vasopressors).

### 2.4. Statistical Analysis

Continuous variables are described as the mean ± SD, and categorical variables are described as the number (%). Missing clinical data were imputed via multiple imputation. Comparisons were performed using the *t*-test or Wilcoxon rank sum test for continuous variables and the chi-square test or Fisher's exact test for categorical variables. Then, univariate and multivariate logistic regression were used to identify risk factors associated with in-hospital mortality for trauma patients with sepsis. ROC curves were drawn, and diagnostic efficacies were determined. Finally, a Kattan-style nomogram was constructed with the program “nomolog” in the Stata user-written program for logistic regression models and used as the in-hospital mortality model. A two-tailed test was performed, and *p* < 0.05 was considered statistically significant. All statistical analyses were performed using StataMP 16.0 (Stata Corporation, College Station, Texas).

## 3. Results

**Figure 1:**
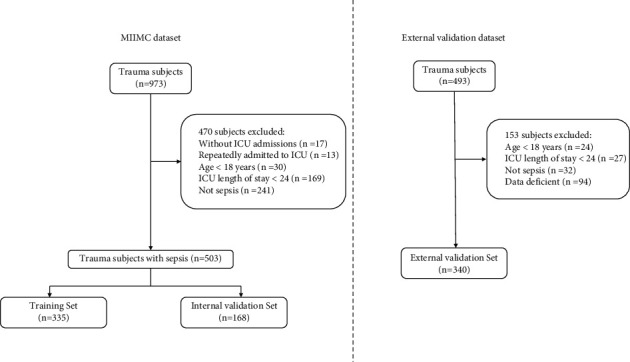
Flow chart of patient enrollment.

For our training and internal validation set, a total of 973 trauma patients who were admitted to the intensive care unit were screened for inclusion, of whom 17 patients were excluded because of repeated admissions to the hospital. A further 30 patients were excluded because they were under 18 years of age, 13 patients without ICU admission were removed. 169 patients had an ICU stay of shorter than 24 hours. 241 patients were also excluded because they did not meet the sepsis criteria. Finally, the remaining 503 patients were eligible for analysis and were randomly divided into a training set (*n* = 335) and a validation set (*n* = 168). The flow chart of patient selection is presented. For the external validation set, a total of 340 patients were finally enrolled in our study (Figure 1). There was no statistically significant difference in demographic characteristics, disease severity score, comorbidity, laboratory outcomes, or interventions between the training set and internal validation set. Besides, the clinical characteristics of the external validation set are also shown in Table 1.

To identify the independent risk factors for in-hospital mortality of trauma patients with sepsis in the ICU, univariable analysis was performed between the survival and nonsurvival groups in the training set, which showed that in-hospital mortality was significantly associated with age, respiratory rate, temperature, and other factors, as shown in Table 2. Then, those characteristics in univariate logistic regression (*p* < 0.05) were incorporated into multiple logistic regression, and the results were reported as odds ratios (95% CI). As shown in Table 3, the results showed that age (1.047 [1.025–1.071]), respiratory rate (1.258 [1.135–1.394]), PTT (1.026 [1.008–1.044]), ventilation (6.703 [1.528–29.408]) and vasopressor use (3.682 [1.502–9.025]) were independent risk factors associated with in-hospital mortality.

**Figure 2:**
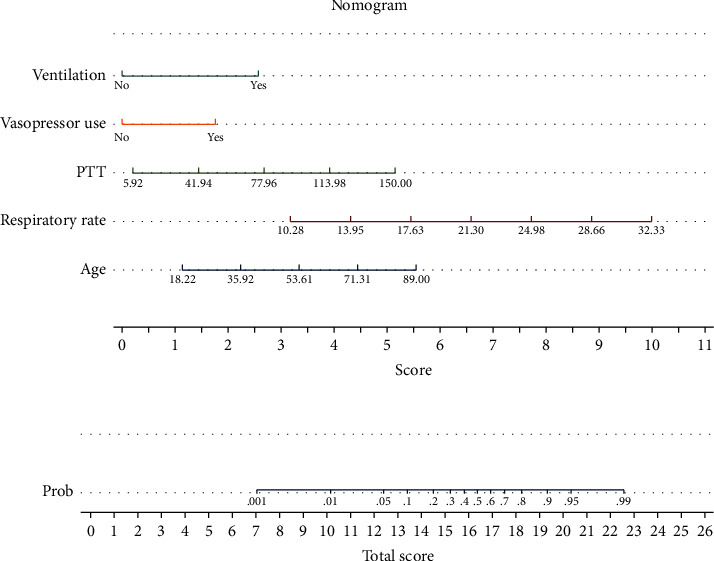
A new nomogram for predicting in-hospital mortality of trauma with sepsis patients.

**Figure 3:**
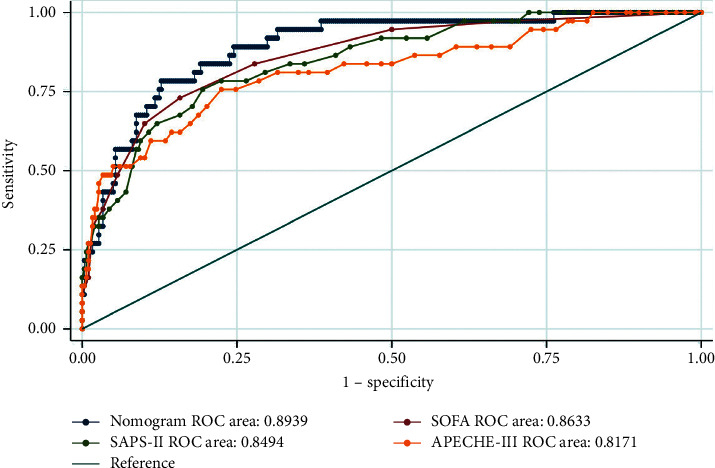
The ROC curve of our nomogram, SOFA score, SAPS-II score, and APECHE-III score in the training set.

**Figure 4:**
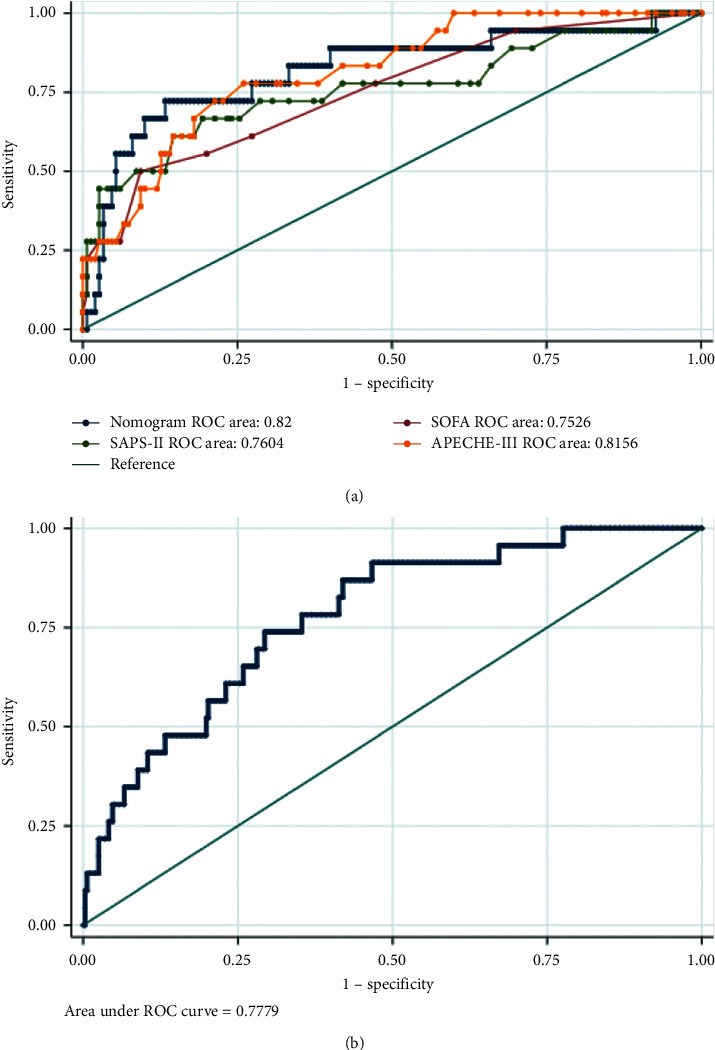
The ROC curve of our nomogram, SOFA score, SAPS-II score, and APECHE-III score in the internal validation set (a) and our nomogram in the external validation set (b).

Finally, a nomogram including the above predictors was established to predict the probability of in-hospital mortality for trauma patients with sepsis (Figure 2). The area under the ROC curve (AUC) of our nomogram was 0.8939, indicating the strong predictive power of the model in the training set; the model showed better accuracy than the SOFA score, SAPS-II score, or APACHE-III score (Figure 3). The nomogram also demonstrated reasonably good accuracy in the internal validation set and external validation set (with AUC values of 0.8200 in the internal validation set and 0.7779 in the external validation set, respectively), as shown in Figure 4.

## 4. Discussion

According to the World Health Organization (WHO), trauma is responsible for 10% of deaths and 16% of disabilities worldwide. Patients with major trauma are prone to septic complications due to the immune dysregulation that occurs after trauma [19]. Sepsis is a life-threatening illness associated with poor prognosis [20]. The incidence of mortality due to post-traumatic sepsis development in the intensive care unit (ICU) is still high, and there is no improvement in outcome in trauma patients with sepsis [2, 21]. Hence, the identification of risk factors for sepsis in patients after trauma is highly important, especially for those in intensive care units. A nomogram was established to predict the probability of in-hospital mortality for trauma patients with sepsis in our study.

In the present study, it was found that age was an independent risk factor. As patients age, decreased immune function and increased comorbidities result in an inability to establish effective and adequate defense mechanisms in the early stages of trauma [22]. Older sepsis patients showed higher levels of immunosuppression and biomarker levels of proinflammation than younger patients [23]. It has long been appreciated that sepsis incidence and in-hospital mortality increase exponentially after age 65 years and age is an independent risk factor of mortality [24]. Overall, age is significant in predicting mortality, which our results also confirmed.

Ventilation is one of the cornerstones of intensive care and one of the most commonly used life support measures for ICU patients. Post-traumatic sepsis patients often develop respiratory failure requiring mechanical ventilation [25]. Our study showed that ventilation was an independent predictor for in-hospital mortality, which may be related to early respiratory failure and later ventilator-induced lung injury.

Respiratory rate is regarded as one of the indicators of systemic inflammatory response syndrome (SIRS). Baek et al. found the SIRS score to be a significant independent factor for in-hospital mortality in multiple trauma patients [26]. A similar conclusion was also found by Napolitano LM [27]. Therefore, the SIRS score may be a useful tool for predicting outcomes associated with in-hospital mortality for trauma patients.

Our results also indicated that vasopressor use was a predictor in trauma patients with sepsis, which was indicative of hypotension and microcirculatory disturbance. The association of increased PTT with in-hospital mortality may be considered representative of trauma-induced coagulopathy. Prolonged partial thromboplastin time is common among trauma patients [28]. Early coagulopathy may be an independent predictor of mortality in trauma patients with sepsis.

In this study, we conclude that the five variables included in our prediction model are independent risk factors for trauma patients with sepsis. Although any single variable could be used for the early prediction of the risk of in-hospital mortality in these patients, there is a limited ability of a single value to predict mortality. When these variables are integrated into a panel, the predictive ability greatly improves. Moreover, the five selected predictors are available within several hours of admission and could establish a more accurate assessment. More importantly, this study is the first to use a nomogram for the in-hospital mortality of trauma patients with sepsis in intensive care units. Patients with a higher risk of in-hospital death may be more likely to receive adequate attention in nursing support and clinical care, which ultimately has a positive impact on patient outcomes [29]. The identification of patients who are at high risk of mortality could also help clinical decision-making regarding appropriate treatment strategies. Last but not least, we found that the new nomogram has higher predictive value compared with the SOFA score, SAPS-II score, and APACHE-III score. Although the above scoring systems are widely used in ICU patients, our nomogram is specific to trauma patients with sepsis receiving intensive care, which may help people better assess the severity and prognosis of these patients.

Our study had some limitations. Some important risk factors, such as blood transfusion and emergency surgery, were not analyzed due to the retrospective nature of this study and because some data were lacking. Although the nomogram was verified with both internal and external validation cohorts, our study did not consider the interaction or nonlinearity for the relationship between covariates and outcome. Hence, the complex relationship between covariates and outcome is unknown in our study. Further machine-learning algorithms might be employed to model the underlying data by using a ground-breaking technique [30]. Finally, our prediction model is static, and it may be more reasonable to mine the association between time series variables and in-hospital mortality for trauma in sepsis patients.

## 5. Conclusion

Our study identified five variables as predictors of in-hospital mortality for trauma patients with sepsis and then derived a nomogram, which could well predict in-hospital mortality for those trauma-related sepsis patients in intensive care units. The nomogram could help clinicians evaluate the condition of the patient and aid in decision-making and patient management, which might have a positive impact on prognosis.

## Figures and Tables

**Table 1:** Baseline characteristics and clinical tests.

Variable	Internal set			External set
	Training set (*n* = 335)	Validation set1 (*n* = 168)	*P* value	Validation set2 (*n* = 340)
Gender, male (%) (n)	68.36 (229)	71.43 (120)	0.481	70.00 (238)
Age (years) (mean ± SD)	50.75 ± 21.81	50.41 ± 20.89	0.866	61.03 ± 18.30
Weight (kg) (mean ± SD)	81.54 ± 22.02	82.85 ± 18.37	0.508	—

Score systems (mean ± SD)				
GCS	13.59 ± 2.65	13.57 ± 2.54	0.927	—
SAPS-II score	31.22 ± 12.45	30.90 ± 12.65	0.784	—
Apache-III score	39.49 ± 17.32	40.96 ± 17.13	0.366	—
SOFA score	4.28 ± 2.41	4.13 ± 2.26	0.486	—

Comorbidity (%) (n)				
Coronary heart disease	4.78 (16)	2.38 (4)	0.195	6.18 (21)
Hypertension	25.37 (85)	22.02 (37)	0.408	17.06 (58)
Diabetes	11.34 (38)	12.50 (21)	0.704	13.53 (46)
COPD	0.6 (2)	0 (0)	0.316	5.88 (20)

Vital signs (mean ± SD)				
Temperature (°C)	37.09 ± 0.61	37.14 ± 0.68	0.451	—
Heart rate (/min)	87.98 ± 16.74	89.11 ± 15.89	0.468	95.26 ± 23.81
Respiratory rate (/min)	18.30 ± 3.96	18.26 ± 3.79	0.932	21.04 ± 6.20
MAP (mmHg)	80.16 ± 9.54	79.46 ± 10.04	0.443	92.16 ± 24.77

Blood routine (mean ± SD)				
WBC (×10^9^/L)	12.98 ± 5.69	12.85 ± 6.44	0.818	15.01 ± 6.42
RBC (×10^9^/L)	3.74 ± 0.71	3.69 ± 0.83	0.506	3.78 ± 0.80
Hemoglobin (g/dL)	11.47 ± 2.20	11.29 ± 2.51	0.411	11.43 ± 24.45
Hematocrit (%)	33.25 ± 6.19	32.99 ± 7.14	0.672	35.28 ± 7.16
Platelet (×10^9^/L)	198.91 ± 101.92	205.88 ± 110.72	0.482	153.91 ± 69.79

Coagulation function (mean ± SD)				
PTT (sec)	31.94 ± 17.88	31.99 ± 15.56	0.975	29.83 ± 11.32
PT (sec)	14.12 ± 2.37	14.25 ± 2.32	0.583	14.75 ± 4.63
INR	1.28 ± 0.29	1.29 ± 0.31	0.830	1.29 ± 0.41

Renal function (mean ± SD)				
BUN (mg/dL)	17.23 ± 12.01	16.25 ± 9.76	0.362	21.96 ± 15.19
Creatinine (mg/dL)	0.89 ± 0.33	0.94 ± 0.51	0.283	0.99 ± 1.03

Electrolyte (mean ± SD)				
Potassium (mEq/L)	4.06 ± 0.62	4.05 ± 0.69	0.880	3.59 ± 0.67
Sodium (mEq/L)	139.44 ± 4.23	139.74 ± 4.32	0.462	138.72 ± 4.70
Chloride (mEq/L)	107.13 ± 5.50	107.54 ± 5.19	0.415	103.35 ± 5.33
Calcium (mg/dL)	8.08 ± 0.94	8.05 ± 0.99	0.811	8.58 ± 0.67
Magnesium (mg/mL)	1.70 ± 0.36	1.65 ± 0.33	0.170	1.94 ± 0.35
Phosphorous (mg/mL)	3.60 ± 1.09	3.42 ± 1.15	0.101	3.18 ± 1.51

Arterial blood gas (mean ± SD)				
Glucose (mg/dL)	154.43 ± 65.44	149.30 ± 54.23	0.382	168.95 ± 109.52
Lactate (mmol/L)	2.64 ± 1.87	2.65 ± 1.66	0.934	3.02 ± 2.67

Treatment first day (%) (n)				
Ventilation	70.45 (236)	72.62 (122)	0.612	70.29 (239)
CRRT	0.3 (1)	1.2 (2)	0.220	2.65 (9)
Vasopressor use	23.28 (78)	21.43 (36)	0.639	35.88 (122)

In-hospital mortality (%) (n)	11.04 (37)	10.71 (18)	0.911	6.76 (23)

**Table 2:** Univariate logistic regression analysis of in-hospital mortality in the training set.

Variable	Survival (*n* = 298)	Nonsurvival (*n* = 37)	*P* value
Gender, male (%) (*n*)	69.13 (206)	62.16 (23)	0.390
Age (years) (mean ± SD)	49.44 ± 21.37	61.33 ± 22.74	0.002
Weight (kg) (mean ± SD)	81.93 ± 22.61	78.41 ± 16.47	0.360

Score systems (mean ± SD)			
GCS	13.70 ± 2.35	12.65 ± 4.26	0.022
SAPS-II score	29.21 ± 10.60	47.43 ± 14.36	<0.001
Apache-III score	36.64 ± 13.80	62.41 ± 24.59	<0.001
SOFA score	3.85 ± 1.92	7.76 ± 3.10	<0.001

Comorbidity (%) (*n*)			
Coronary heart disease	4.36 (13)	8.11 (3)	0.314
Hypertension	25.84 (77)	21.62 (8)	0.578
Diabetes	11.74 (35)	8.11 (3)	0.511
COPD	0.34 (1)	2.70 (1)	0.078

Vital signs (Mean ± SD)			
Temperature (°C)	37.14 ± 0.56	36.73 ± 0.82	<0.001
Heart rate (/min)	88.14 ± 15.96	86.71 ± 22.30	0.625
Respiratory rate (/min)	17.92 ± 3.74	21.34 ± 4.44	<0.001
MAP (mmHg)	80.08 ± 9.40	80.82 ± 10.76	0.656

Blood routine (mean ± SD)			
WBC (×10^9^/L)	12.95 ± 5.75	13.23 ± 5.30	0.775
RBC (×10^9^/L)	3.73 ± 0.68	3.75 ± 0.93	0.933
Hemoglobin (g/dL)	11.47 ± 2.10	11.47 ± 2.90	0.996
Hematocrit (%)	33.24 ± 5.83	33.34 ± 8.70	0.932
Platelet (×10^9^/L)	200.39 ± 101.04	187.00 ± 109.47	0.452

Coagulation function (mean ± SD)			
PTT (sec)	30.01 ± 13.05	47.43 ± 35.84	<0.001
PT (sec)	13.98 ± 2.10	15.25 ± 3.77	0.002
INR	1.26 ± 0.27	1.42 ± 0.43	0.002

Renal function (mean ± SD)			
BUN (mg/dL)	16.60 ± 11.46	22.32 ± 15.01	0.006
Creatinine (mg/dL)	0.92 ± 0.48	1.09 ± 0.64	0.051

Electrolyte (mean ± SD)			
Potassium (mEq/L)	4.04 ± 0.59	4.21 ± 0.83	0.098
Sodium (mEq/L)	139.29 ± 4.02	140.68 ± 5.52	0.060
Chloride (mEq/L)	106.83 ± 5.10	109.54 ± 7.71	0.005
Calcium (mg/dL)	8.08 ± 0.84	8.05 ± 1.53	0.833
Magnesium (mg/mL)	1.69 ± 0.35	1.77 ± 0.39	0.199
Phosphorous (mEq/L)	3.52 ± 0.99	4.18 ± 1.64	<0.001

Arterial blood gas (mean ± SD)			
Glucose (mg/dL)	150.29 ± 64.81	187.73 ± 61.57	0.001
Lactate (mmol/L)	2.56 ± 1.72	3.26 ± 2.73	0.031

Treatment first day (%) (*n*)			
Ventilation	67.79 (202)	91.89 (34)	0.002
CRRT	0.34 (1)	0 (0)	0.724
Vasopressor use	18.12 (54)	64.86 (24)	<0.001

**Table 3:** Multivariate analysis of independent predictors.

Risk factors	OR	95% *CI*	*P* value
Age (years)	1.047	1.025–1.071	<0.001
Vasopressor use	3.682	1.502–9.025	0.004
Ventilation	6.703	1.528–29.408	0.012
Respiratory rate (/min)	1.258	1.135–1.394	<0.001
PTT (sec)	1.026	1.008–1.044	0.004

## Data Availability

The data analyzed in this paper are available upon request to the corresponding author.
